# A systematic review of ego depletion phenomenon in group-based hierarchical relations

**DOI:** 10.3389/fpsyg.2025.1569692

**Published:** 2025-12-11

**Authors:** Funda Genç Turhan, Muharrem Ersin Kuşdil

**Affiliations:** Department of Psychology, Bursa Uludağ University, Bursa, Türkiye

**Keywords:** ego depletion, resource depletion, intergroup relations, group-based hierarchical relations, self-control, social dominance theory

## Abstract

**Introduction:**

This systematic review examines the relationship between intergroup relations and ego depletion, aiming to understand how self-control resources are affected in social hierarchies.

**Methods:**

A total of 35 peer-reviewed articles and four unpublished doctoral dissertations were reviewed. Findings were categorized based on the social position of the target person’s group in intergroup interactions.

**Results:**

Intergroup contacts depleted the self-control resources of dominant group members when they were required to suppress biases. For subordinate group members, engaging in intergroup interactions, witnessing bias, or facing bias threats also depleted self-control resources. Depleted dominant members exhibited increased prejudice and stereotyping, whereas depleted subordinate members showed lower perception of prejudiced behaviors and higher liking for dominant group members.

**Discussion:**

The findings are interpreted within the framework of social dominance theory, highlighting how ego depletion influences intergroup relations and reinforcing the role of group-based hierarchies in shaping social behavior.

## 1 Introduction

This review aims to examine the role of ego depletion in the context of intergroup relations, with a particular focus on how self-regulatory processes shape and are shaped by power dynamics and social hierarchies. Individuals’ ability to control their emotions, thoughts and behaviors plays a critical role in both their personal success and social interactions (Baumeister and Tierney, 2012; Moffitt et al., 2011). This capacity for self-regulation, however, is not unlimited and can become depleted, which can lead to decreased performance on subsequent tasks (Baumeister et al., 1998). This phenomenon, called ego depletion, can affect individuals’ performance on social and cognitive tasks. The ego depletion effect was replicated in various alternative self-control tasks (Muraven et al., 1998), sparking significant interest in ego depletion in social psychology (Cunningham and Baumeister, 2016). The concept of ego depletion has also faced several criticisms, with some scholars questioning its validity. Critics have pointed to replication failures (such as Hagger et al., 2016; Englert and Bertrams, 2021), the ease with which the effect can be mitigated (Friese and Wänke, 2014), the lack of a clear operational definition of self-control and the use of questionable self-regulation tasks (Lurquin and Miyake, 2017) as reasons for skepticism. Etherton et al. (2018), in their meta-analysis study, reported that the research results provided strong evidence for the null hypothesis. Based on these doubts, some researchers have taken a pessimistic view (Vadillo, 2019), suggesting that the study of ego depletion will disappear by 2020.

Dang (2016), on the other hand, argued that the failures reported in Hagger et al.’s (2016) meta-analysis might be linked to the type of manipulation employed and unsuccessful manipulations of the ego depletion. Subsequent research has supported this view, showing that stronger manipulations continue to reveal the effect (Baumeister et al., 2024). Dang et al. (2021), through a preregistered multilab replication, demonstrated a small but significant effect of ego depletion. More recent evidence suggests that even 45 minutes of self-control effort can lead to increased aggression due to sleep-like frontal brain activity (Ordali et al., 2024). Current analyses of the ego depletion effect have also highlighted several important methodological factors. First, longer and more intense manipulations tend to produce stronger depletion effects. Additionally, the task assigned to the control group is crucial; if the control task is boring, it may induce depletion on its own (Baumeister et al., 2024). Therefore, to clearly observe the ego depletion effect, the tasks given to the depletion and control groups must differ not only in difficulty but also in terms of monotony. In general, despite the pessimistic stance, interest in the ego depletion effect has persisted well beyond 2020.

Some scholars have emphasized that refining rather than abandoning the concept may yield valuable insights (Blázquez et al., 2017; Inzlicht and Friese, 2019). Several researchers have highlighted the importance of cognitive and motivational processes in explaining the effects of ego depletion, shifting the focus from a resource model to cognitive and motivational perspectives (e.g., Inzlicht and Schmeichel, 2012; Kool and Botvinick, 2014; Kurzban et al., 2013). The ego depletion effect, for example, is defined by Inzlicht and Schmeichel (2012) as a brief shift in motivation and focus toward other areas following the application of self-control to a particular task. This effect is further explained by the opportunity cost model of Kurzban et al. (2013). Their model states that people become less inclined to continue self-regulation work after they have been doing it for a while because they are more conscious of other fulfilling activities they may do in its place.

Over time, the theory of self-resources has evolved from a simple model of resource depletion to one that emphasizes resource conservation. Building on this shift, current research is increasingly investigating the real-world implications of ego depletion, including its role in various social and behavioral processes and the factors that moderate its impact (e.g., Baumeister et al., 2024; Miyake and Carruth, 2023; Rehren, 2024). Research on the ego depletion effect in real-world situations highlights the crucial role of self-regulatory processes in interpersonal relationships. Recent studies have indicated that ego depletion mediates the relationship between ostracism and negative risk-taking behavior (Chen et al., 2024). Other line of research has examined ego depletion in organizational settings, revealing that factors such as workaholism and abusive leadership can drain self-regulatory resources (Jin, 2023). One significant area where the implications of ego depletion have been studied is in sports. Researchers have explored the effects of ego depletion among various groups of athletes (e.g., Dorris et al., 2012; Furley et al., 2013). Englert (2017) published a review article highlighting the importance of self-control as a key factor influencing physical performance. Most recently, a meta-analysis of 12 studies found that ego depletion significantly reduces athletes’ sports performance (Yan et al., 2025).

Despite a large body of research on ego depletion, however, systematic reviews combining these studies are relatively rare. Some reviews focus on theoretical aspects of ego depletion (e.g., Baumeister and Vohs, 2007; Friese et al., 2019), while others explore its application in fields like sports (Englert, 2017) and auditing (Hurley, 2015). The only systematic review on ego depletion in specific groups focused on university students, suggesting that they are particularly susceptible to depletion due to various emotional and cognitive factors (Gissubel et al., 2018). Unlike the previous reviews of ego depletion, this present review focuses explicitly on the interplay between ego depletion and intergroup relations. By situating ego depletion within the context of intergroup relations, this review seeks to clarify its implications for understanding systemic inequalities. Intergroup relations are well-suited for testing depletion because they involve clear group boundaries, frequent self-regulatory demands, and enduring power hierarchies that may amplify depletion effects. Differences between groups tend to be pronounced, as the contrast between the experimental and control groups is often strong. Moreover, intergroup relations frequently include hierarchical dynamics, allowing for long-lasting and intensified manipulations.

The aim of this review is not just to revisit the debate over the existence of ego depletion but to emphasize its potential role in explaining real-world phenomena. Specifically, rather than treating ego depletion as an isolated effect, we focus on its implications within intergroup relations, particularly regarding the maintenance of social hierarchies. By situating ego depletion in this context, we seek to clarify how variations in self-regulatory capacity may influence and be influenced by power dynamics and interactions across social groups. Research shows that ego depletion reduces individuals’ ability to control their prejudices and biases (Ma et al., 2013; Zhang and Peng, 2020). While individuals are normally able to suppress prejudicial thoughts to conform to social norms, when self-resources are depleted, this control weakens, and prejudicial attitudes and behavioral biases can emerge easily. These findings suggest that ego depletion affects not only cognitive performance but also the perception of fairness and equality in social interactions.

This review addresses two critical research questions (RQ): (1) How intergroup interactions influence the self-regulatory processes of individuals? and (2) How ego depletion, in turn, shapes the outcomes of intergroup interactions? At first glance, answers to these questions seem contradictory: While some studies have reported that the experience of ego depletion exacerbates prejudicial attitudes (e.g., Ma et al., 2013; Zhang and Peng, 2020), others have shown that depletion tends to have mitigating consequences in intergroup interactions (Koslov, 2010). A closer look reveals that these variations are deeply rooted in whether an individual belongs to a subordinate or dominant group, shaping both the antecedents and consequences of ego depletion. These distinctions are critical in sustaining and reinforcing hierarchical structures, demonstrating how self-regulatory processes can perpetuate systemic inequalities. Based on this observation, these findings can be examined separately for dominant and subordinate group members through the lens of social dominance theory (SDT), a prominent theory in social psychology that suggests societies are structured based on group-based hierarchies, and these hierarchies are sustained not through constant conflict but via societal consensus (Sidanius and Pratto, 1999).

### 1.1 Social dominance theory

SDT posits that most societies are structured around three fundamental group-based hierarchies. The first of these is the age system in which older adults exert unequal power over younger adults. The second is the gender system, where men have unequal power over women. The third group-based hierarchy includes arbitrary–set categories. These categories refer to the socially constructed groupings and may include any categories, such as race, ethnicity, and other categories (Pratto et al., 2006). A central assumption of the theory (Sidanius and Pratto, 1999) is that dominant groups hold many material and symbolic resources associated with a positive social value (e.g., political power, wealth, protection by force, diverse and delicious food, suitable housing, and sanitation). On the other hand, Sidanius and Pratto (1999) argue that subordinate groups are left to cope with many problems associated with negative social value (e.g., unemployment, diseases, dangerous and low-status jobs, housing in adverse conditions, poor health care, bad food, and poverty).

SDT provides a comprehensive framework for understanding how group-based hierarchies are established and perpetuated in societies. Hierarchy is a fundamental characteristic of social groups and intergroup relations and the longevity of it depends significantly on the extent to which they are legitimized within a given society. As one of the main concepts in SDT, legitimizing myths serves as powerful tools for reinforcing inequality by shaping collective attitudes, societal beliefs, values, and stereotypes that either uphold or undermine existing hierarchies (Pratto et al., 2006; Sidanius and Pratto, 1999). At the individual level, Social Dominance Orientation (SDO)—a person’s inclination to support or oppose group-based inequalities—plays a critical role in sustaining hierarchical relationships (Bernardo, 2013; Hoyt and Simon, 2016; Pratto et al., 1994; Pratto et al., 2006).

Although SDT does not detail the psychological mechanisms behind behavioral asymmetries, ego depletion might play a role. It is possible to expect that both dominant and subordinate group members are susceptible to ego depletion in different ways. Subordinate group members, frequently confronted with negative stereotypes, may experience a depletion of self-resources, exacerbating self-debilitating behaviors. In contrast, dominant group members, shielded by favorable stereotypes, are less affected or may even experience enhanced self-regulation. However, in societal contexts where hierarchy-reducing myths are more prevalent among individuals, dominant group members may feel compelled to suppress prejudices, which also requires significant self-control. This dynamic potentially leads to ego depletion among dominant group members when interacting with subordinates under such conditions (Crandall et al., 2002; Webster et al., 2014). Examining ego depletion in an intergroup context may provide a psychological mechanism for testing the basic assumptions and concepts of SDT.

SDT further emphasizes that the actions and attitudes of both dominant and subordinate group members contribute to maintaining these hierarchies. For instance, institutional discrimination initiated by dominant groups forms a structural basis for inequality (Sidanius and Pratto, 1999; Pratto et al., 2006). Meanwhile, subordinate groups may engage in self-debilitating behaviors—such as absenteeism, neglecting responsibilities, or substance abuse—that reinforce their disadvantaged positions (Pratto et al., 2006) and stereotypes about them. Research illustrates that low academic performance among African American and Latino students is often linked to such behaviors, including higher absenteeism, reduced homework time, and decreased attention in class (Pratto et al., 2006). On the other hand, dominant group members benefit from stereotypes, which act as legitimizing myths to enhance their performance. For example, the stereotype lift and stereotype boost phenomena suggest that stereotypes favoring dominant groups can positively impact their performance (Shih et al., 2012; Walton and Cohen, 2003).

From this perspective, ego depletion’s effects on prejudice and bias can be seen as a crucial mechanism for maintaining social hierarchies. When the self-regulatory resources of dominant groups are depleted, they are more likely to respond with prejudice. In contrast, subordinate groups must exert more self-regulation to prevent ongoing prejudice and discrimination. This dynamic helps sustain social pressures and hierarchies.

### 1.2 Key terms

To avoid conceptual ambiguity in the subsequent sections of this review, it is essential to first provide clear definitions of the terms employed. Although the terms ‘self-control’ and ‘self-regulation’ are often used interchangeably in literature, it is also useful to highlight the subtle differences between them. Self-regulation refers to the processes by which individuals control their emotions, thoughts, and behaviors in line with their goals such as suppressing impulses, directing attention, or behaving in accordance with social norms. Self-control, on the other hand, is defined as a narrower dimension of self-regulation. It generally involves suppressing immediate impulses, habits, or automatic reactions, such as refraining from eating unhealthy food or remaining calm in times of anger are examples of self-control (Baumeister and Vohs, 2004; Muraven and Baumeister, 2000). In this review, the term *self-regulation* will be used, as it encompasses attempts at self-control as well.

The functioning of these processes is closely related to the concept of self-resources. Self-resources are the psychological capacities that enable self-regulation and self-control; attention, willpower, and cognitive energy are the primary elements of these resources. Because these resources are assumed as limited, they can be temporarily depleted after intensive use. In this context, ego depletion refers to the negative impact of previous self-regulatory attempts on subsequent self-regulatory activities. In studies examining the ego depletion effect, participants in the experimental group are typically administered a manipulation requiring self-regulation or self-control, after which participants in both the experimental and control groups are expected to perform a different task (Baumeister et al., 1998; Muraven et al., 1998). The tasks used for measurement and manipulation of the ego depletion vary across studies. In some studies, tasks require control of the dominant response, such as the Stroop test or “e-marking,” while other tasks relate to everyday behaviors, such as aggression or emotional reactions, or the use of cognitive resources, such as math or mental arithmetic (Hagger et al., 2016).

The literature emphasizes that ego depletion is distinct from cognitive depletion, which refers to a general decrease in cognitive resources. Nevertheless, research indicates that these two phenomena are closely interconnected, demonstrating that attempts to self-regulate may also impair cognitive abilities (Schmeichel, 2007; Schmeichel et al., 2003). Indeed, some studies have assigned participants to cognitively demanding tasks or measured their cognitive performance afterward (e.g., Carter et al., 2015; Park et al., 2008). As Robinson et al. (2010) pointed out that ego depletion should be understood not only as the depletion of resources, but also through the cognitive control mechanisms. In this review, we prefer using the term *ego depletion*, as a subtype of *resource depletion*, encompassing both the depletion of resources in self-regulatory processes and the broader effects of cognitive depletion.

### 1.3 Hypotheses derived from the basic assumptions of SDT

**Figure 1:**
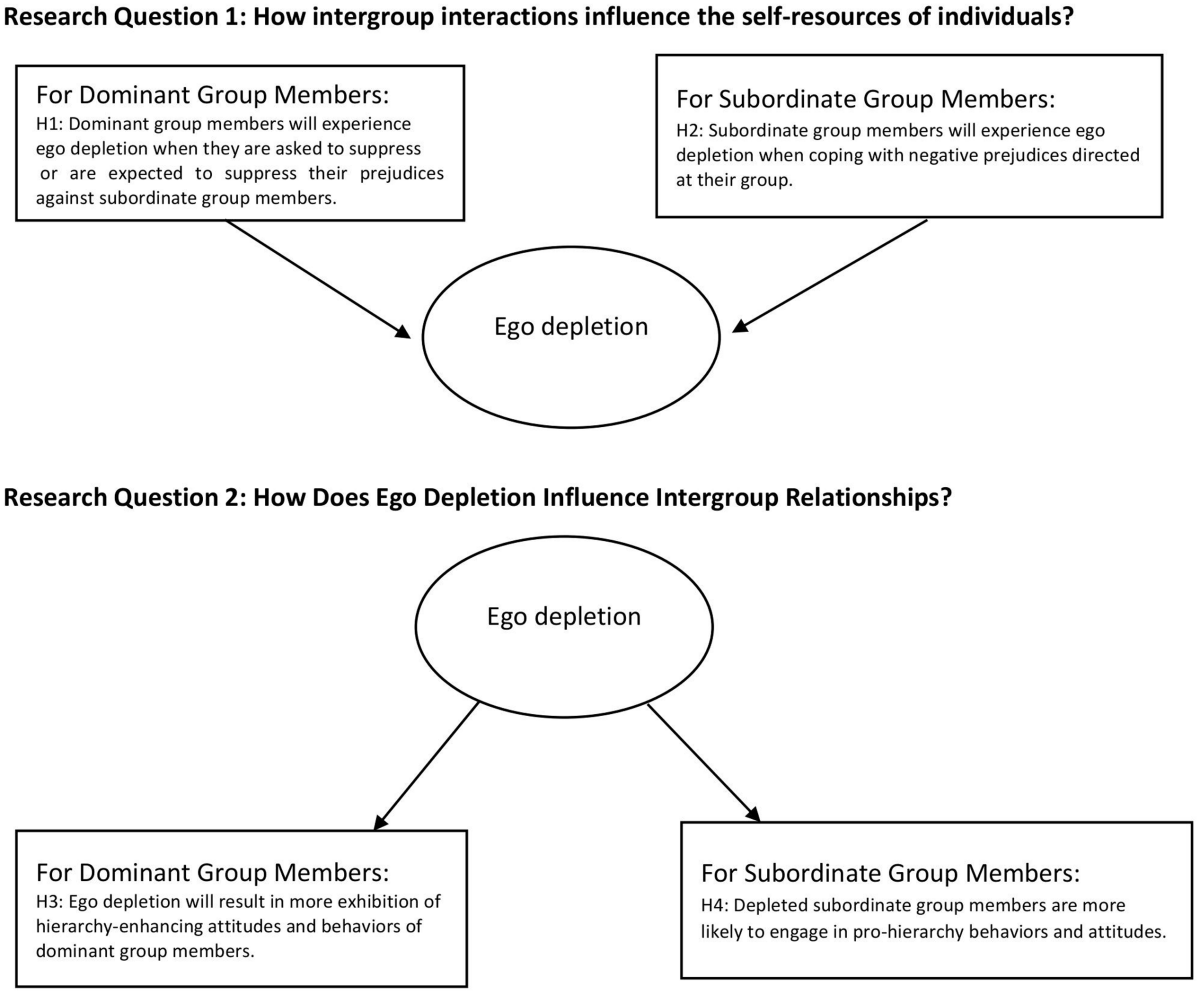
Conceptual overview of intergropu interactions and self-resources in the literature.

We propose that the discrepancies in existing findings may be linked to the status of the group to which an individual belongs within the social hierarchy. Based on the basic assumptions of SDT, we articulated hypotheses (See Figure 1) related to our research questions that may help clarify these findings. This article explores the interactions between self-resources and intergroup relations through the lens of SDT and aims to strengthen the connections between key concepts to create a more holistic framework.

#### 1.3.1 Hypotheses for the RQ1: How intergroup interactions influence the self-resources of individuals?

SDT states that members of dominant groups are driven to maintain social hierarchies. In these hierarchical relationships, dominant group members do not always need to self-regulate since the system promotes their perceived dominance. Conflict occurs when people are urged to repress their preconceptions. On the one hand, they are driven to legitimize the hierarchy; on the other, they face egalitarian standards that contradict this incentive (Pratto et al., 2006). This internal conflict can lead to the use of cognitive resources, raising the load of self-regulation and diminishing one’s own resources.

Hypothesis 1: Dominant group members will experience ego depletion when they are asked to suppress or are expected to suppress their prejudices against subordinate group members.

Members of subordinate groups, on the other hand, frequently encounter unfavorable biases against their group (Pratto et al., 2006). They have to deal with the threats these biases pose. Ego depletion is a result of the constant effort needed to manage the emotional and cognitive burden of bias directed at them.

Hypothesis 2: Subordinate group members will experience ego depletion when coping with negative prejudices directed at their group.

#### 1.3.2 Hypotheses for the RQ2: How does ego depletion influence intergroup relations?

In general, dominant group members have a strong tendency to maintain existing hierarchies, which leads them to endorse hierarchy-enhancing myths and to demonstrate attitudes and behaviors that support hierarchy, such as stereotypes, prejudice, and discriminatory biases (Pratto et al., 2006). Even when they attempt to suppress or hide these tendencies under certain conditions, doing so requires self-regulation. Consequently, depletion of self-regulatory resources weakens their ability to control these hierarchy-enhancing attitudes and behaviors.

Hypothesis 3: Ego depletion will result in more exhibition of hierarchy-enhancing attitudes and behaviors of the dominant group members.

Typically, subordinate group members may resist or seek equality due to their disadvantaged position. However, when they experience depletion, they lose the self-regulatory capacity to resist the hierarchy. In this situation, they may become more reliant on internalized hierarchy-enhancing ideologies (e.g., system justification or the belief that “higher groups are better”). As a result, depletion increases the tendency of subgroup members to conform to and accept the hierarchy.

Hypothesis 4: Depleted subordinate group members are more likely to engage in pro-hierarchy behaviors and attitudes.

## 2 Method

### 2.1 Search strategies

To achieve the stated objectives, a comprehensive literature search was conducted using EBSCOhost, PubMed, and Web of Science with the keywords “intergroup interaction,” “stereotype,” and “prejudice,” each combined with terms of “ego depletion” and “resource depletion.” The survey spans roughly 30 years, covering the period from 1998—when the concept of ego depletion was first introduced—to February 2022. This review adhered to the PRISMA guidelines (Preferred Reporting Items for Systematic Reviews and Meta-Analyses), recognized as international standards for systematic reviews and meta-analyses (Moher et al., 2015).

### 2.2 Inclusion criteria

**Figure 2:**
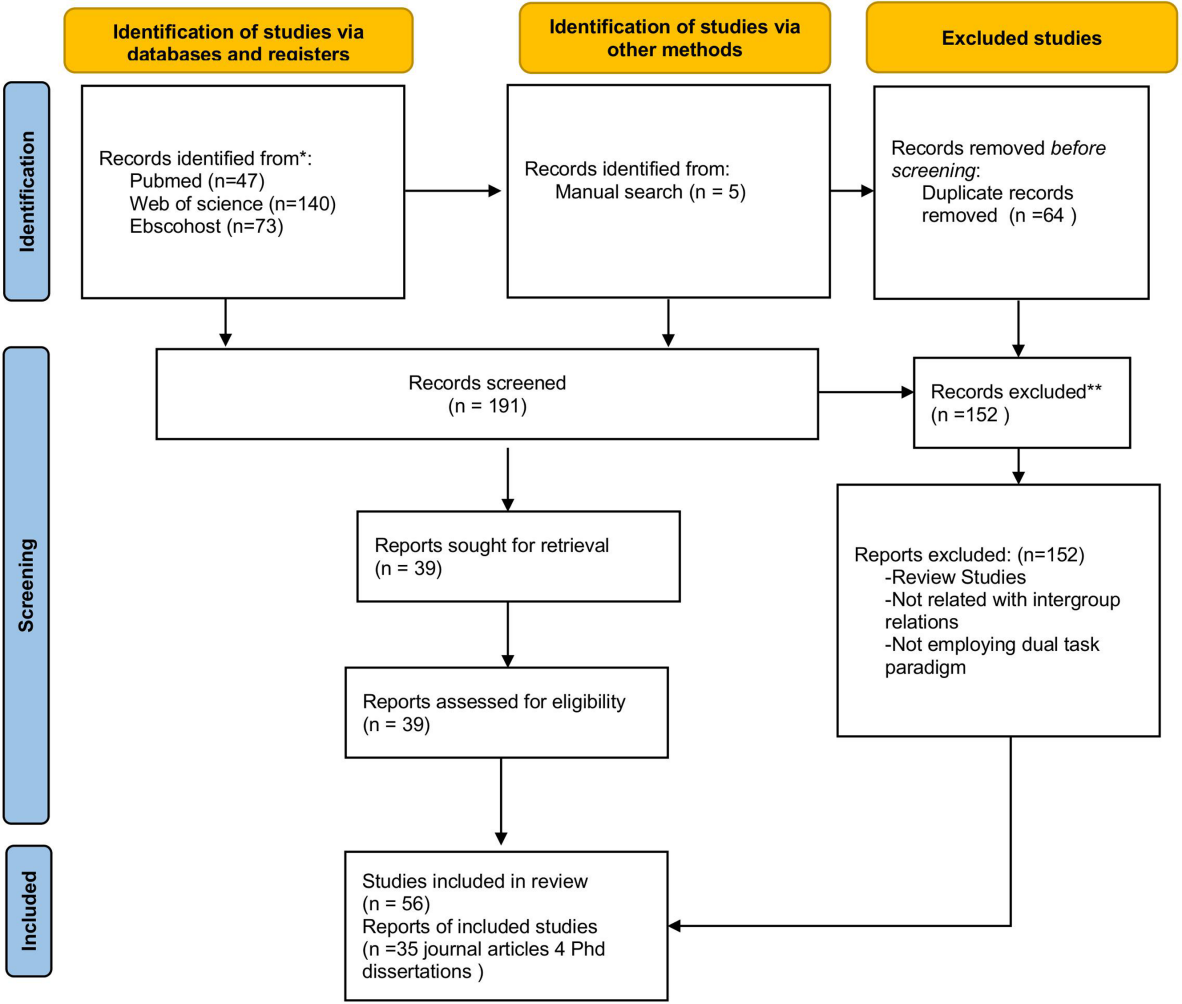
PRISMA 2022 FLOW diagram for new systematic reviews which includes searches of databases, registers and other sources.

Studies were included in the present review if they (a) employed an experimental or quasi-experimental research design, (b) examined ego depletion within the framework of the dual-task paradigm—that is, involving a manipulation task followed by a measurement task, which could take one of two forms: (i) studies examining the effects of group interactions on ego depletion, and (ii) studies employing ego depletion manipulations followed by assessments of group interactions or related outcomes—and (c) included participants or targets from either dominant or subordinate social groups, or both. Based on these criteria, 39 research reports (35 journal articles and four unpublished doctoral dissertations, comprising a total of 56 studies) were retained for analysis out of the 65 initially identified (see Figure 2 for the PRISMA flowchart).

## 3 Results

The findings are organized into two main sections to address the study’s main RQs: (1) How do intergroup relations affect self-resources? and (2) how does ego depletion influence intergroup relationships? The first section explores the effects of intergroup dynamics on the self-control resources of dominant and subordinate group members. The second section presents findings of from the relevant studies on how ego depletion impacts attitudes and behaviors during intergroup interactions. Following the SDT’s explanations on the set systems, women, Black individuals, LGBTQ+ individuals, and facially stigmatized individuals are categorized as subordinate groups in this review. In contrast, majority groups, such as men and White individuals, are categorized as the dominant group members in this review.

### 3.1 How do intergroup interactions influence self-resources?

This section presents findings on how intergroup interaction depletes self-resources. First, it examines dominant group members, the factors influencing this effect, and the results. Then, it answers how and when intergroup interactions lead to ego depletion in subordinate group members.

#### 3.1.1 Basic characteristics of the studies

The results of 41 (from these, two studies are investigated for both dominant and subordinate group members) studies are included in this section. Approximately half these studies (*N* = 22) included participants of subordinate groups, whereas 21 studied the depleting effects of intergroup contacts among dominant group members. Over half (*N* = 22) of the research looked at how various races interacted with one another. Five of the studies examined people with varied sexual orientations, while nine of the studies focused on relationships between different genders. Participants from arbitrary established categories (having facial stigma, skin-head persons, groups depending on the department of study) were included in the remaining experiments.

Researchers in the included studies used diverse methods to evaluate ego depletion and self-regulation processes. The methodologies utilized to assess ego depletion and self-regulation processes differed between investigations. In the majority of the studies (N = 30), ego depletion was evaluated using the Stroop task (Stroop, 1935). Four of the studies assessed behavioral signs of impaired self-control by evaluating impulsive purchases, aggressive conduct, eating, and hazardous decisions. One study of the studies assessed cognitive resources using IQ tests for both dominant and subordinate groups Five of the studies utilized an anagram problem, and one study used a well-known physical self-control challenge, squeezing a handgrip. Finally, one research utilized the Weapon Identification Task (Payne, 2001), which demands self-control. supplementary Table 1 provides detailed summaries of the research examined in this section.

**Table 1:** Studies on the depleting effects of intergroup relations.

Depletion type	Reference	Country	Sample	Group status	Group	Intergroup interaction manipulation	Measurement task	Main findings	Effect size	df
Ego depletion	Richeson and Shelton (2003)	USA	50 White students (21 girls, 29 boys)	Dominant group	Race	Face-to-face interaction with a Black or White experimenter	Stroop task	The level of prejudice was found to be associated with a decrease in following interracial interaction, but not following same-race interaction.	Not reported	Not reported
Ego depletion	Richeson et al. (2003)	USA	Study 1: 15 White students (8 girls)	Dominant group	Race	Face-to-face interaction with Black experimenter	Stroop task	White participants with higher implicit prejudice showed greater impairment in Stroop performance after interactions with Black experimenters.	*r* = 0.67	Study 1: 13
Ego depletion	Richeson et al. (2003)	USA	Study 2: 15 White students (8 girls)	Dominant group	Race	Face-to-face interaction with a Black or White experimenter	Stroop task	The correlation between implicit prejudice and Stroop performance after same-race contacts was not significant.	*r* = −0.07	Study 2:13
Ego depletion	Richeson and Trawalter (2005)	USA	Study 1: 60 White students (40 girls)	Dominant group	Race	Face-to-face interaction with a Black or White collaborator	Stroop task	Study 1: Increasing the self-control requirements of the interview resulted in greater Stroop interference.	partial η^2^ = 0.15	Study 1: 52
Ego depletion	Richeson and Trawalter (2005)	USA	Study 2: 64 White students (41 girls)	Dominant group	Race	Face-to-face interaction with a Black or White collaborator	Stroop task	Study 2: Reducing the self-control requirements of the interview resulted in a decrease in Stroop interference.	η^2^ ≈ 0.13	Study 2: 28
Ego depletion	Richeson and Trawalter (2005)	USA	Study 3: 68 White students (47 girls)	Dominant group	Race	Face-to-face interaction with a Black or White collaborator	Stroop task	Study 3: Participants who engaged in interracial interactions exhibited greater Stroop interference compared to those who interacted with a same-race partner. However, when participants were allowed to misattribute their anxiety to the environment, this impairment was eliminated.	partial η² = 0.18	Study 3: 32
Ego depletion	Trawalter and Richeson (2006)	USA	45 White girl students	Dominant group	Race	Face-to-face interaction with Black collaborator	Stroop task	The intergroup interview impaired Stroop performance for both prevention-focused and control participants, whereas promotion-focused participants were protected from this effect.	*r* = 0.29	41
Ego depletion	Gaillot et al. (2007)	USA	Study 4: 53 students (42 women)	Dominant group	Sexual orientation	Suppressing stereotypes	Stroop task	However, this effect disappeared after two weeks of self-control practice.	η^2^p ≈ 0.10	Study 4: 48
Ego depletion	Salvatore and Shelton (2007)	USA	250 participants (122 Black, 128 White)	Dominant group	Race	Witnessing the bias	Stroop task	White subjects showed significantly more interference in the blatant-prejudice condition than in the other two prejudice conditions.	η2 = 0.08	58
Ego depletion	Madera and Hebl (2012)	USA	Study 1: 171 participants (60 men, 111 women)	Dominant group	Facial Stigma Scar	Looking at the picture on the screen	Stroop task	Participants who saw a stigmatized applicant performed worse on the Stroop task than the control group	η2 = 0.20	Study 1:168
Ego depletion	Madera and Hebl (2012)	USA	Study 2:38 full-time managers (21 women, 17 men)	Dominant group	Facial Stigma Scar	Conducting face-to-face structured interviews	Stroop task	Managers who interviewed people with facial scars performed poorly on the Stroop task compared to the control group.	η^2^ = 0.12	Study 2: 36
Ego depletion	Pearson et al. (2013)	USA	84 White students (60% female)	Dominant group	Race	Face-to-face interaction with Black female collaborator	Stroop task	In interactions between White and Black individuals, participants with high implicit bias demonstrated poorer Stroop performance when suppressing negative emotions. In contrast, those with low bias showed reduced performance when suppressing positive emotions.	Not reported	71
Ego depletion	Zabel et al. (2015)	USA	67 White students (39 female, 28 male)	Dominant group	Race	Making a video email conversation with a Black or White collaborator	Stroop task	Negative impact of meeting with a Black person on Stroop performance occurred only when the conservation involved intimate topics.	η^2^ = 0.06	63
Ego depletion	Peter-Hagene (2016)	USA	197 participants	Dominant group	Race	Evaluation of defendant	Stroop task	Mixed juries were significantly more depleted than jurors in all-White juries.	Partial η^2^ = 0.03	181
Ego depletion	Gordijn et al. (2004)	Holland	Study 1: 51 students	Dominant group	Skinhead people	Study 1: Suppressing stereotypes when describing a skinhead person	self-control effort ratings	Participants asked to suppress stereotypes reported greater self-control effort than the control group, and this effect was stronger among those low in internal motivation to avoid prejudice.	Cohen’s *d* ≈ 0.90	Study 1: 47
Ego depletion	Gordijn et al. (2004)	Holland	Study 2: 52 students	Dominant group	Skinhead people	Study 2: Suppressing stereotypes when describing a skinhead person	Solving anagrams	Participants who were asked to suppress their stereotypes performed worse on a subsequent anagram-solving task; however, this effect was only observed in those with low internal motivation to suppress these stereotypes.	η^2^ ≈ 0.06	Study 2: 76
Ego depletion	Gaillot et al. (2007)	USA	Study 1: 40 students (15 women)	Dominant group	Sexual Orientation	Study 1: Suppressing stereotypes	Solving anagrams	People with low motivation to avoid prejudice performed poorly when asked to avoid stereotypes while describing the homosexual person. Two weeks of self-control practices eliminated this effect.	η^2^ ≈ 0.19	34
Ego depletion	Gaillot et al. (2007)	USA	Study 2: 104 students (70 women)	Dominant group	Sexual Orientation	Study 2: Suppressing stereotypes	Solving anagrams	People with low motivation to avoid prejudice performed poorly when asked to avoid stereotypes while describing the homosexual person. However, this effect was weaker among those who had recently engaged in more self-control behaviors	*d* ≈ 0.41	98
Ego depletion	Gaillot et al. (2007)	USA	Study 3: 179 students (93 women)	Dominant group	Sexual Orientation	Study 3: Suppressing stereotypes	Solving anagrams	People with low motivation to avoid prejudice performed poorly when asked to avoid stereotypes while describing the homosexual person. Two weeks of self-control practices eliminated this effect.	*d* ≈ 0.38	170
Ego depletion	Amodio (2009)	USA	40 participants (72% female)	Dominant group	Race	Face to face interaction	Weapon identification task	In the case of interviewing the Black interviewer, Participants reported more anxiety with a Black interviewer, and only those with higher cortisol reactivity underperformed on the Weapon Identification Task.	*r* = 0.55	33
Ego depletion	Berry (2015)	USA	Study 2: 57 students (39 female)	Dominant group	Sexual Orientation	Suppressing stereotypes	Impulsive buying	Participants who were asked to suppress stereotypes stated more impulsive buying desires.	η^2^ = 138	45
Cognitive depletion	Wetter (2015)	USA	39 White females	Dominant group	Race	Testing	Intelligence test	White female participants underperformed on the verbal portion of the intelligence test when it was administered by Black researchers compared to when it was administered by a White researcher.	η^2^ ≈ 0.19	34
Ego depletion	Inzlicht et al. (2006)	USA	Study 2: 21 Black, 21 White students	Subordinate group	Race	Study 2: Stereotype threat	Stroop task	Black participants exposed to stereotypical threat underperformed on the cognitive self-control task.	*d* = 0.84	Study 2: 36
Ego depletion	Richeson et al. (2005)	USA	60 Black (38 male) students	Subordinate group	Race	Video call	Stroop task	Black participants interacting the White partner performed poorly on the Stroop task compared to Black participants interacting the Black partner. As the implicit positive attitudes of Black participants towards Whites increased, this effect decreased.	*r* = −0.44	28
Ego depletion	Salvatore and Shelton (2007)	USA	250 students (122 Black, 128 White)	Subordinate group	Race	Witnessing the bias	Stroop task	Black subjects exhibited greater interference in the ambiguous-prejudice condition compared to the other two prejudice conditions.	η2 = 0.05	82
Ego depletion	Johns et al. (2008)	USA	Study 2: 46 female students	Subordinate group	Gender	Stereotype threat	Stroop task	Exposure to stereotype threats resulted in poor performance on the Stroop task and math tests. The tendency to suppress anxiety contributed to this effect. The negative consequences of stereotype threat disappeared when a reappraisal style was used instead of anxiety suppression.	*d* = 0.82	41
Ego depletion	Carr and Steele (2010)	USA	Study 2b:56 students(31 female, 25 male)	Subordinate group	Gender	Stereotype threat	Stroop task	Ego depletion mediated the relationship between stereotype threat and risky decision making.	*d* = 0.92	Study 2b = 29
Ego depletion	Bair and Steele (2010)	Canada	78 Black students (62 female)	Subordinate group	Race	Face to face interaction	Stroop task	Black participants high in race centrality showed greater Stroop impairment after interacting with a White collaborator, especially under racist conditions.	*d* = 0.63	61
Ego depletion	Babbitt and Sommers (2011)	USA	Study 1: 53 interracial; 23 White/White participant pairs	Subordinate group	Race	Face to face interaction	Stroop task	Only Black participants in socially focused interracial interactions showed reduced Stroop performance compared to White participants in the same interaction condition.	*r* = 0.24	Study 1: 80
Ego depletion	Holoien and Shelton (2012)	USA	158 students: (31 White/White; 25 White/Asian; 23 White/Black participant)	Subordinate group	Race	Face to face interaction	Stroop task	Ethnic minorities experienced greater ego-depletion when contacted with White participants who were quickly shown color-blind messages prior to interaction, compared to those shown multicultural messages.	*d* ≈ 0.58	71
Ego depletion	Murphy et al. (2012)	USA	Study 1: 43 Black students	Subordinate group	Race	Face to face interaction	Stroop task	Black participants who were exposed to subtle bias exhibited greater cognitive depletion, as indicated by higher Stroop interference, compared to those exposed to blatant bias or no bias at all. This finding suggests that subtle forms of racial bias are especially disruptive to cognitive control.	η^2^ = 0.11	Study 1: 40
Ego depletion	Murphy et al. (2012)	USA	Study 2: 59 Latino students	Subordinate group	Race	Interaction	Stroop task	Latino participants who encountered subtle bias demonstrated greater cognitive depletion, as indicated by higher Stroop interference scores, compared to those who experienced blatant bias.	η^2^ = 0.10	Study 2:56
Ego depletion	Stahl et al. (2012)	Holland	Study 1: 63 social science students	Subordinate group	Study department	Stereotype threat	Stroop task	Stereotype threat temporarily improved cognitive control, supporting the resource recruitment hypothesis.	η^2^p = 0.13	Study 1: 61
Ego depletion	Stahl et al. (2012)	Holland	Study 2: 108 social science students	Subordinate group	Study department	Stereotype threat	Stroop task	Stereotype threat enhances cognitive control only with prevention focus or no specific focus, but not under promotion focus.	η^2^p = 0.06	Study 2: 101
Ego depletion	Stahl et al. (2012)	Holland	Study 3: 163 female students	Subordinate group	Study department	Stereotype threat	Stroop task	When focused on prevention, stereotype threat initially boosts math performance but leads to decreased performance after extended exposure due to resource depletion. In contrast, a promotion focus shows no significant effect.	η^2^p = 0.08	Study 3: 150
Ego depletion	Banks (2015)	USA	65 Black female student	Subordinate group	Race	Face to face interaction	Stroop task	Stroop performance of Blacks exposed to microaggression by a White researcher decreased significantly, while participants in the control group did not.	*R*^2^ = 0.08	62
Ego depletion	Inzlicht and Kang (2010)	Canada	Study 4: 52 students	Subordinate group	Gender	Stereotype threat	Stroop task	Women who experienced stereotype threat demonstrated reduced cognitive control; however, using reappraisal techniques helped protect against this negative effect.	*d* = 0.98	Study 4: 39
Ego depletion	Inzlicht et al. (2006)	USA	Study 3: 61 female students	Subordinate group	Gender	Studies 3: Stereotype threat	Squeezing the handle	Participants exposed to stereotype threat underperformed on physical self-control tasks.	*d* = 0.66	Study 3: 54
Ego depletion	Walker et al. (2022)	USA	131 female students	Subordinate group	Gender	Implicit and explicit discrimination	Stroop task	Female participants exposed to subtle (implicit) discrimination performed worse on task performance measures than control participants, and this effect was partially mediated by ego depletion.	η^2^p = 0.12	121
Ego depletion	Inzlicht and Kang (2010)	Canada	Study 1: 34 female students	Subordinate group	Gender	Stereotype threat	Aggression	Dealing with stereotype threat reduces self-control and increases aggression, and these effects are not explained by mood or frustration.	*d* = 0.79	Study 1: 28
Ego depletion	Inzlicht and Kang (2010)	Canada	Study 2: 49 female students	Subordinate group	Gender	Stereotype threat	Eating ice cream	Coping with stereotype threat depleted resources, leading to increased overeating, particularly among women who are highly aware of stigma.	*d* = 1.06	Study 2:41
Ego depletion	Inzlicht and Kang (2010)	Canada	Study 3: 118 students (72% female)	Subordinate group	Gender	Stereotype threat	Choosing	Recalling a social identity threat depleted self-control resources, increasing reliance on automatic, risky decision-making, independent of mood or general negative affect.	χ^2^ = 6.19	Study 3:1
Cognitive depletion	Wetter (2015)	USA	39 White female students	Subordinate group	Gender	Strereotype threat	Intelligence test	No significant main effect of the stereotype threat condition was observed on any of the WAIS-IV subtests	η^2^p = 0–0.06	35

#### 3.1.2 Risk of bias assessment

**Figure 3:**
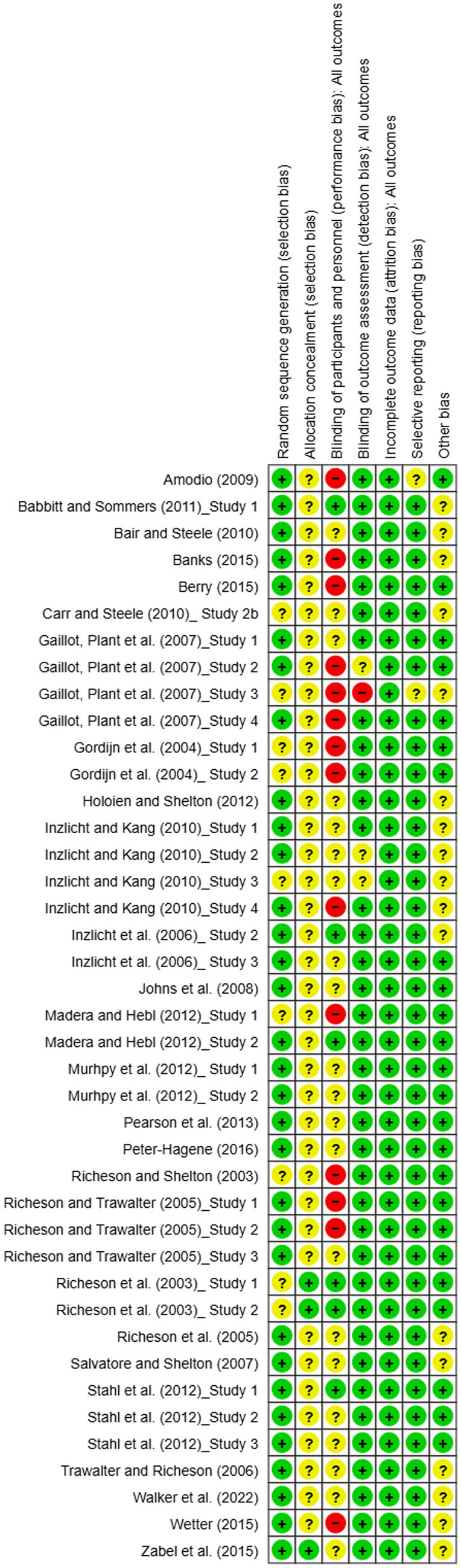
Risk of bias summary for studies on the depleting effects of intergroup relations.

**Figure 4:**
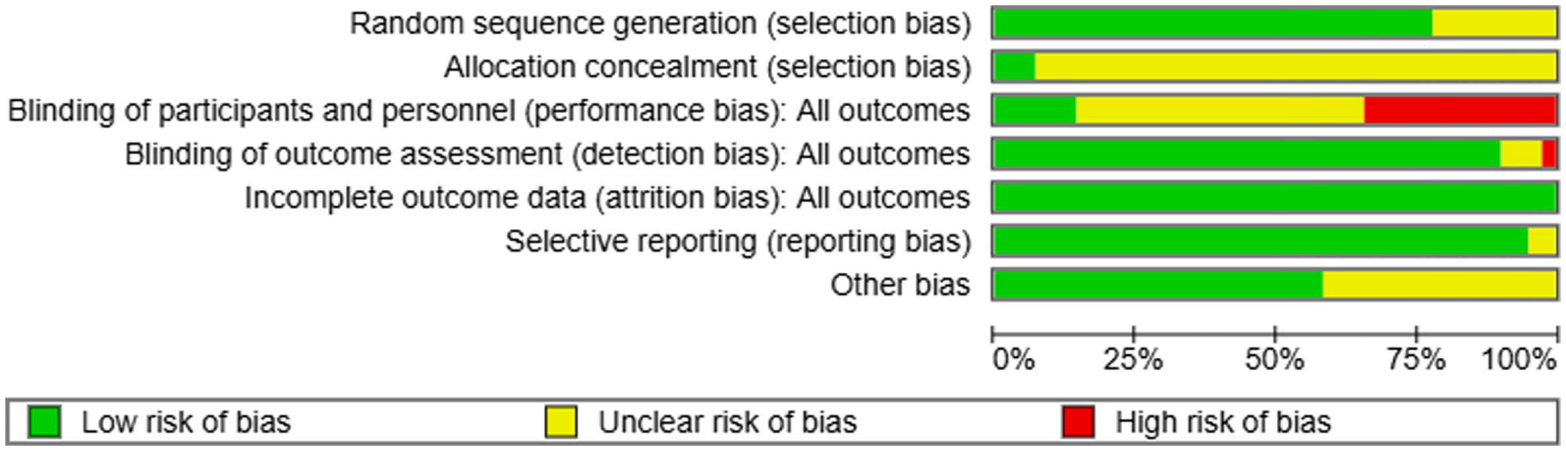
Risk of bias graph for studies on the depleting effects of intergroup relations.

In this review, we evaluated 41 studies—comprised of 23 journal articles and three PhD theses—to assess the risk of bias. Among the 41 studies, 32 reported the use of random assignment. However, most did not ensure that the allocation of participants was adequately concealed. While selective reporting, completeness of outcome data, and other potential sources of bias were generally deemed to pose a low risk, the majority of studies exhibited a risk of bias related to blinding of participants and personnel. Overall, all 41 studies demonstrated some potential bias issues based on our risk assessment. Detailed results can be found in Figures 3, 4.

#### 3.1.3 The depleting effects of intergroup interactions on dominant group members’ self-resources

When dominant group members interact with subordinate group members, they frequently experience ego depletion, according to a thorough review of numerous studies. In situations where they must repress stereotypes or observe the prejudices of others (Gordijn et al., 2004; Salvatore and Shelton, 2007), or when they deal directly with those who have face stigma (Madera and Hebl, 2012), this depletion may be evident. According to research, this ego depletion might result in more impulsive behaviors (Berry, 2015) and decreased cognitive capacities (Wetter, 2015).

Individual characteristics influence the outcomes of these interactions. Richeson and Shelton (2003) found that both implicit and explicit prejudices among White participants positively correlated with lower self-regulatory performance after interviews with Black partners. Similarly, Trawalter and Richeson (2006) showed that participants instructed to avoid prejudice during interracial interactions experienced greater performance decrements on subsequent self-control tasks compared to control groups or participants who were told to interact only positively.

Another important factor is the Motivation to Avoid Prejudice (MAP), which refers to how strongly individuals are driven to prevent expressing prejudiced thoughts or behaviors (Plant and Devine, 1998). Research indicates that individuals with low MAP experience greater ego depletion when required to suppress stereotypes (Gordijn et al., 2004; Gaillot et al., 2007).

Physiological responses also play a role in this process. For example, elevated cortisol levels following interracial interactions are linked to poorer performance on self-control tasks, suggesting that stress may act as a mechanism that depletes self-resources (Amodio, 2009).

In addition, the dynamics and composition of the interaction significantly impact outcomes. Interactions that involve socially focused and affiliative questions require more self-regulatory resources than those that are merely task-focused or do not involve such questions (Babbitt and Sommers, 2011; Zabel et al., 2015). These results indicate that egalitarian contexts intensify the conflict between hierarchy-maintaining motivations and egalitarian expectations, thereby increasing the burden on self-regulatory resources. Moreover, working with mixed-race groups (e.g., mixed-jury groups) demands more regulatory resources than working with homogeneous groups (Peter-Hagene, 2016). These findings imply that perceived threats to group-based status or superiority heighten regulatory demands and accelerate ego depletion.

Overall, these findings suggest that intergroup interactions result in ego depletion for dominant group members, depending on context. The interplay of individual characteristics, motivational states, physiological stress, and the nature of the interactions shapes the extent of this ego depletion effect. Thus, the current body of research partially supports the hypothesis (H1) that intergroup interactions do not inherently deplete the self-resources of dominant group members. Studies examining the content of interviews (Babbitt and Sommers, 2011; Zabel et al., 2015) provide evidence that interactions with subordinate groups do not always lead to ego depletion.

Aligning with Hypothesis 1, considerable empirical evidence indicates that the suppression of prejudice is fundamental driver of ego depletion in dominant group members. Research consistently shows that participants who are asked to inhibit bias during interracial interactions perform worse on subsequent self-control tasks, reflecting the cognitive costs of suppression (Richeson and Shelton, 2003; Trawalter and Richeson, 2006). The depleting effect on self-resources from efforts to avoid prejudice is particularly evident in a study where self-control requirements in interviews were manipulated (Richeson and Trawalter, 2005). It is not the contact itself that depletes resources; rather, it is the regulatory demands within these interactions—such as suppressing prejudice, controlling stereotypes, or managing egalitarian expectations—that contribute to ego depletion. While SDO has not been directly examined in the reviewed studies, related research indicates that both explicit and implicit prejudice predict greater depletion after intergroup interactions (Richeson and Shelton, 2003; Richeson et al., 2003). Therefore, although further empirical work is necessary to clarify the direct role of SDO, existing findings provide a plausible basis for this hypothesis.

To summarize, these findings suggest that intergroup interactions do not consume self-regulatory resources directly. Instead, they become depleting under conditions that require prejudice suppression, compliance with egalitarian norms, or management of status threats. Additionally, individual differences, such as levels of prejudice, appear to moderate the extent of depletion. This synthesis highlights the importance of considering both situational and dispositional factors to understand the psychological costs of intergroup interactions.

#### 3.1.4 Depleting effects of intergroup relationships on subordinate group members’ self-resources

Intergroup interactions often include significant cognitive and self-regulatory challenges for members of subordinate groups, typically to a greater extent than those experienced by dominant group members. One central mechanism underlying this effect is stereotype threat—the concern with confirming negative stereotypes about one’s group (Steele and Aronson, 1995). Stereotype threat has been shown to tax self-regulatory resources, producing declines in self-control performance (Inzlicht et al., 2006; Johns et al., 2008). Supporting this account, Inzlicht and Kang (2010) demonstrated that participants exposed to stereotype threat exhibited poor self-control performance, as reflected in EEG activity during a math test. Carr and Steele (2010) further found that ego depletion mediated the relationship between stereotype threat and decision-making, with women displaying heightened risk aversion and loss aversion under threat. Beyond decision-making, stereotype threat has been linked to more aggressive behavior, increased consumption of unhealthy food, and greater willingness to take risks (Inzlicht and Kang, 2010).

Not all studies, however, have replicated these findings. Wetter (2015), for instance, found no evidence that stereotype threat impaired performance on verbal comprehension, working memory, or perceptual reasoning tasks. This null effect was attributed to participants’ weak identification with the tested domain and to the reliance on IQ measures rather than tasks such as the Stroop, which are commonly used in stereotype threat research. Such results suggest that the effects of stereotype threat on self-regulation may depend on both methodological choices and the degree of domain identification. An important aspect of this study is that White female students experienced stereotype threat. However, the impact of this stereotype threat manipulation might be limited or absent in this sample due to factors such as low identification with the domain or the specific types of tasks used.

The depletion of self-regulatory resources is not limited to stereotype threat but also emerges from exposure to bias in intergroup settings. Salvatore and Shelton (2007) found that Black participants experienced greater interference in ambiguous prejudice situations, whereas White participants were more disrupted by blatant prejudice. Murphy et al. (2012) similarly showed that subtle bias during interviews with White collaborators produced greater cognitive impairment among Black and Latino participants than either blatant or no bias. Extending this work, Walker et al. (2022) identified ego depletion as the mechanism through which subtle discrimination impaired subsequent performance.

Racial micro aggressions have comparable effects: Banks (2015) found that Black women exposed to micro aggressions from a White researcher showed significant declines in self-regulation. Several individual and situational factors moderate these outcomes. For example, Black participants with more positive implicit attitudes toward White individuals were less affected by intergroup interviews than those with stronger in-group favoritism (Richeson et al., 2005).

Rejection sensitivity has also been linked to self-control impairment, with higher stigma sensitivity predicting lower regulatory capacity (Inzlicht et al., 2006). Identity centrality is another key moderator: Black participants for whom race was central to their self-concept experienced greater depletion following exposure to racism (Bair and Steele, 2010).

In terms of motivational orientation, regulatory focus has been found to alter how stereotype threat drains resources. Under prevention focus, participants initially showed enhanced self-control, but performance later declined as resources were exhausted; no such effect was observed under promotion focus (Stahl et al., 2012).

Finally, the cues communicated by dominant-group members can significantly shape outcomes. Holoien and Shelton (2012) reported that ethnic minorities interacting with White individuals primed with “color-blind” messages experienced more depletion than those paired with Whites primed with a “multicultural ideology.” Similarly, the content of the interaction matters: socially oriented intergroup exchanges impaired Black participants’ Stroop performance, whereas task-focused exchanges did not (Babbitt and Sommers, 2011).

Taken together, these findings indicate that intergroup interactions can deplete the self-regulatory resources of subordinate group members, but the extent of this depletion is shaped by both contextual factors (e.g., type of bias, dominant-group ideology, interaction content) and individual differences (e.g., domain identification, racial identity centrality, regulatory focus).

The scope of research with subordinate group members is relatively broad and complex. Apart from one-to-one exposure to prejudice in intergroup interactions, stereotype threat alone can be sufficient for ego depletion in subordinate group members. Although most of the studies on stereotype threat (Inzlicht et al., 2006; Johns et al., 2008) showed that exposure to stereotype threat leads to a decrease in the self-control performance of subordinate group members, Wetter (2015) could not obtain such a result. This might be due to failures in experimental procedures (stereotype threat variation or self-regulation measurement). Wetter (2015) cited several factors, including the fact that participants did not identify with the domain in which they were assessed, as to why the stereotype threat did not reach the level necessary to have an impact. Additionally, the results may have been impacted by the fact that the self-control measures utilized in this study (IQ test, Wetter, 2015) were distinct from commonly used measuring techniques (Stroop task) in other studies.

Prejudice (mostly subtle prejudice) exhibited by the dominant group members also negatively affects the self-resources of subordinate group members (Bair and Steele, 2010; Murphy et al., 2012; Banks, 2015). In addition, Salvatore and Shelton (2007), in their study on witnessing bias in job interviews, supported the negative effect of blatant prejudice by demonstrating that Black participants had the most significant impairment on self-resources in the subtle prejudice condition.

Overall, the findings support Hypothesis 2: intergroup interactions in hierarchical contexts place significant self-regulatory burdens on subordinate group members. Research on stereotype threat suggests that the expectation or experience of prejudice reduces self-regulatory capacity (Inzlicht et al., 2006; Johns et al., 2008). Similarly, ambiguous prejudices, microaggressions, or biased interview contexts also negatively impact self-control and performance (Salvatore and Shelton, 2007; Murphy et al., 2012; Banks, 2015).

Research indicates that contextual factors can exacerbate ego depletion. For instance, social interactions consume more cognitive resources than task-focused interactions (Babbitt and Sommers, 2011). Similarly, directing dominant group members with “color-blind” messages increases depletion in subordinate group participants, whereas “multicultural” messages can mitigate this effect (Holoien and Shelton, 2012).

Individuals whose racial identity is central (Bair and Steele, 2010) or who are highly sensitive to stigma (Inzlicht et al., 2006) experience greater ego depletion when exposed to prejudice. Likewise, members closely tied to their group exhibit increased depletion in such contexts (Richeson et al., 2005). Further, interactions framed with multicultural or egalitarian messages buffer against these negative effects more effectively than color-blind messages (Holoien and Shelton, 2012), suggesting that contextual norms actively shape the degree to which subordinate group members experience depletion.

To conclude, stereotype threat and prejudice deplete subordinate group members’ self-resources. However, the magnitude of this effect is closely linked to the nature of the context, the form of the prejudice, and individual differences (e.g., identity centrality and stigma sensitivity). While most studies support our hypotheses, differing results, such as those found by Wetter (2015), highlight the need for methodological diversity.

### 3.2 How does ego depletion influence intergroup relationships?

The relationship between self-control and intergroup interactions is two-sided: Just as intergroup relations affect people’s self-resources, decreases in self-control can also affect individuals’ intergroup behaviors and attitudes. This section presents the effects of ego depletion on intergroup interactions.

#### 3.2.1 Basic characteristics of the studies

This section summarizes the findings from a total of 15 studies. Thirteen of these studies examined ego depletion among dominant group members, while only two focused on subordinate group members. Most of the studies included participants from various racial groups (*N* = 11), with one study involving different age groups (*N* = 1) and three studies including participants of various sexual orientations (*N* = 3).

Methods used to manipulate ego depletion varied across the studies. Most employed well-established tasks typically used in ego depletion research, including the Stroop task, E-marking task, story writing task, and emotion regulation task (*N* = 11). Meanwhile, four studies utilized tasks that required broader cognitive resources, such as Mental Mathematics and the Attention Network Task. Furthermore, one research used the glucose hypothesis—which postulates that self-control depends on glucose as a finite physiological resource—to investigate the idea of ego depletion (Gailliot et al., 2007; Gailliot and Baumeister, 2007). This study investigated whether the loss of self-regulatory resources might be explained by changes in glucose levels. Detailed descriptions of the studies reviewed in this section can be found in Table 2.

**Table 2:** Studies on the ego depletion effect on intergroup relations.

Depletion type	Reference	Country	Sample	Group status	Group	Manipulation	Measurement of Stereotype/Prejudice	Main findings	Effect size	Df
Ego depletion	Govorun and Payne (2006)	USA	72 students [Mostly male (62.5%) and White (84.7%)]	Dominant group	Race	Stroop Task	Weapon identification task	Participants who are depleted show more bias when their automatic stereotype activation is high. While self-resource depletion did not affect automatic processes, it negatively affected controlled processes.	*d* ≈ 0.49	70
Ego depletion	Zhang and Peng (2020)	China	65 students (23 women)	Dominant group	Age	Stroop task	Frequency of using stereotypes	Depleted participants rated older adults as more forgetful compared to participants in the control group.	*d* = 0.55	63
Ego depletion	Ma et al. (2013)	USA	Study 1: 77 students: 40 males, 36 females, 1 unspecified; 44 White, 14 Asian, 10 Latino, 5 Black, 1 unspecified	Dominant group	Race	Stroop task	Shooting task	Study 1: Ego-depletion led to greater racial bias in shooting decisions, especially toward Black targets.	ηp^2^ = 0.05	Study 1:75
Ego depletion	Muraven (2008)	USA	Study 1: 56 White students	Dominant group	Race	Marking the letters E	Frequency of using stereotypes	Participants who were highly motivated to avoid prejudice used more positive and fewer stereotypic descriptors for African Americans when they were not depleted, but showed increased negative stereotyping when they were depleted.	η^2^p ≈ 0.095	48
Ego depletion	Buzinski and Kitchens (2017)	USA	Study 4: 92 students [70 women, mostly White (83%) and heterosexual (97.8%)]	Dominant group	LGBT	Marking the letters E	Study 4: Amount of hand cleaner used	Depleted participants used more hand sanitizer after being provided with LGBT-related information (a sign of disgust). On the other hand, this effect did not occur under the high social pressure to be unbiased.	η^2^ = 0.05	Study 4 = 87
Ego depletion	Legault et al. (2009)	Canada	Study 2: 135 White students (99 female, 36 male)	Dominant group	Race	Marking the letters E	Study 2: Implicit Association Test	Ego- depletion resulted in increased prejudice in individuals who were not motivated to avoid prejudice.	η^2^p = 0.03	66
Ego depletion	Tadmor et al. (2018)	China	Study 2: 111 Israeli Jewish participants	Dominant group	Race	Story writing task	Study 2: Supporting stereotypes	Participants who were not depleted showed lower endorsement of Arab stereotypes after recalling a multicultural experience, whereas depleted participants showed no reduction.	*r* = 0.21	107
Ego depletion	Buzinski and Kitchens (2017)	USA	Study 1: 40 students [15 female, mostly White (90%), and heterosexual (95%)]	Dominant group	LGBT	Story writing task	Study 1: Motivation to avoid bias	Depleted participants showed decreased internal motivation to prevent prejudice against LGBTs.	η^2^ = 0.20	38
Ego depletion	Muraven (2008)	USA	Study 2: 41 White students	Dominant group	Race	Emotion Regulation Task	Frequency of using stereotypes	Participants who were highly motivated to avoid prejudice used fewer negative and more positive traits to describe African Americans when they were not depleted, but showed increased negative stereotyping when they were depleted.	partial η² = 0.11	41
Ego depletion	Park et al. (2008)	USA	124 students (72 women)	Dominant group	Race	Solving anagram	Shooting Task	The effect of depletion on shooting task is larger for the participant with low implicit motivation to control prejudice.	*β* = −0.28	113
Ego depletion	Gailliot et al. (2009)	USA	51 students (25 female, 25 male, 1 unspecified)	Dominant group	Sexual orientation	Increased resource of self-control with provided glucose	Frequency of using stereotypes	If glucose was provided to participants with a high level of prejudice, they used less stereotypical expressions when asked to describe homosexual people.	*d* ≈ 0.95	49
Cognitive depletion	Apfelbaum and Sommers (2009)	USA	82 White students	Dominant group	Race	Attention network task	Perceived bias level	Depleted participants were rated as less biased when interviewed by the Black collaborator compared to control group participants.	*r* = 0.30	78
Cognitive depletion	Koslov (2010)	USA	123 pairs: Black and White students	Dominant group	Race	Mental mathematics	Overcorrection	When cognitive resources were available, White females with high levels of external motivation to control prejudice overcorrected toward their Black partners, but not when they are depleted.	*d* ≈ 0.90	32
Cognitive depletion	Koslov (2010)	USA	123 pairs: Black and White students	Subordinate group	Race	Mental mathematics	Face-to-face interaction	Ego-depletion reduced hypervigilance, leading Black female students with high rejection sensitivity to evaluate their White partners more positively	*d* ≈ 0.73	33
Cognitive depletion	Carter et al. (2015)	USA	115 Black students	Subordinate group	Race	Attention network task	Interaction and message via computer	Partners who behaved in modern racist ways were perceived as less biased by depleted participants compared to the control group.	η^2^p = 0.03	111

#### 3.2.2 Risk of bias assessment

**Figure 5:**
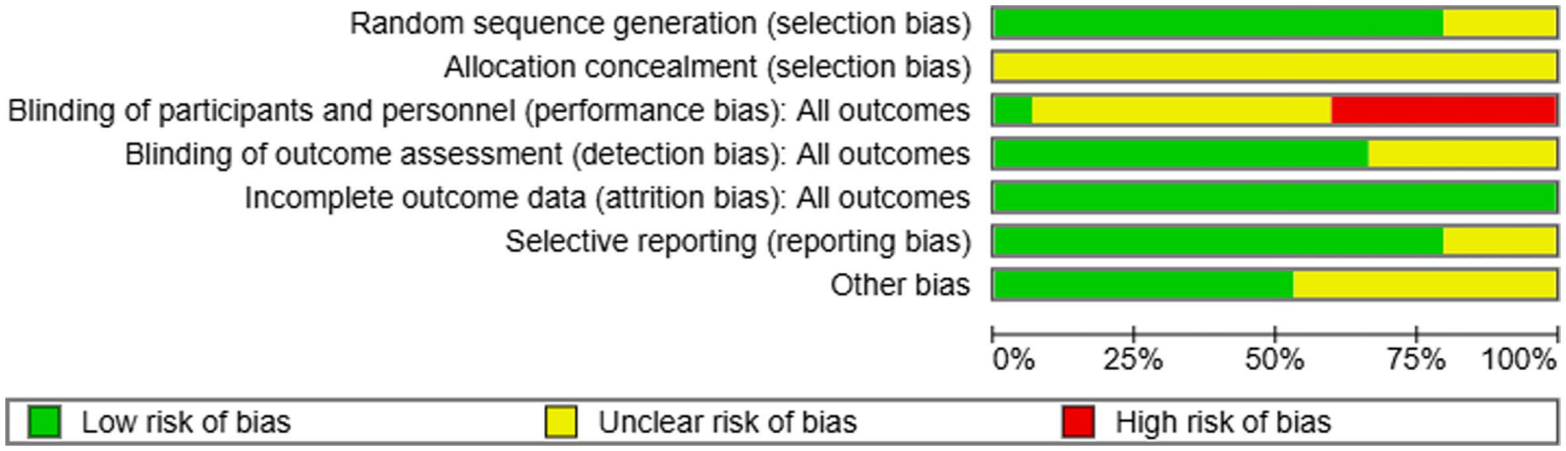
Risk of bias graphs for studies on the ego depletion effect on intergroup relations.

**Figure 6:**
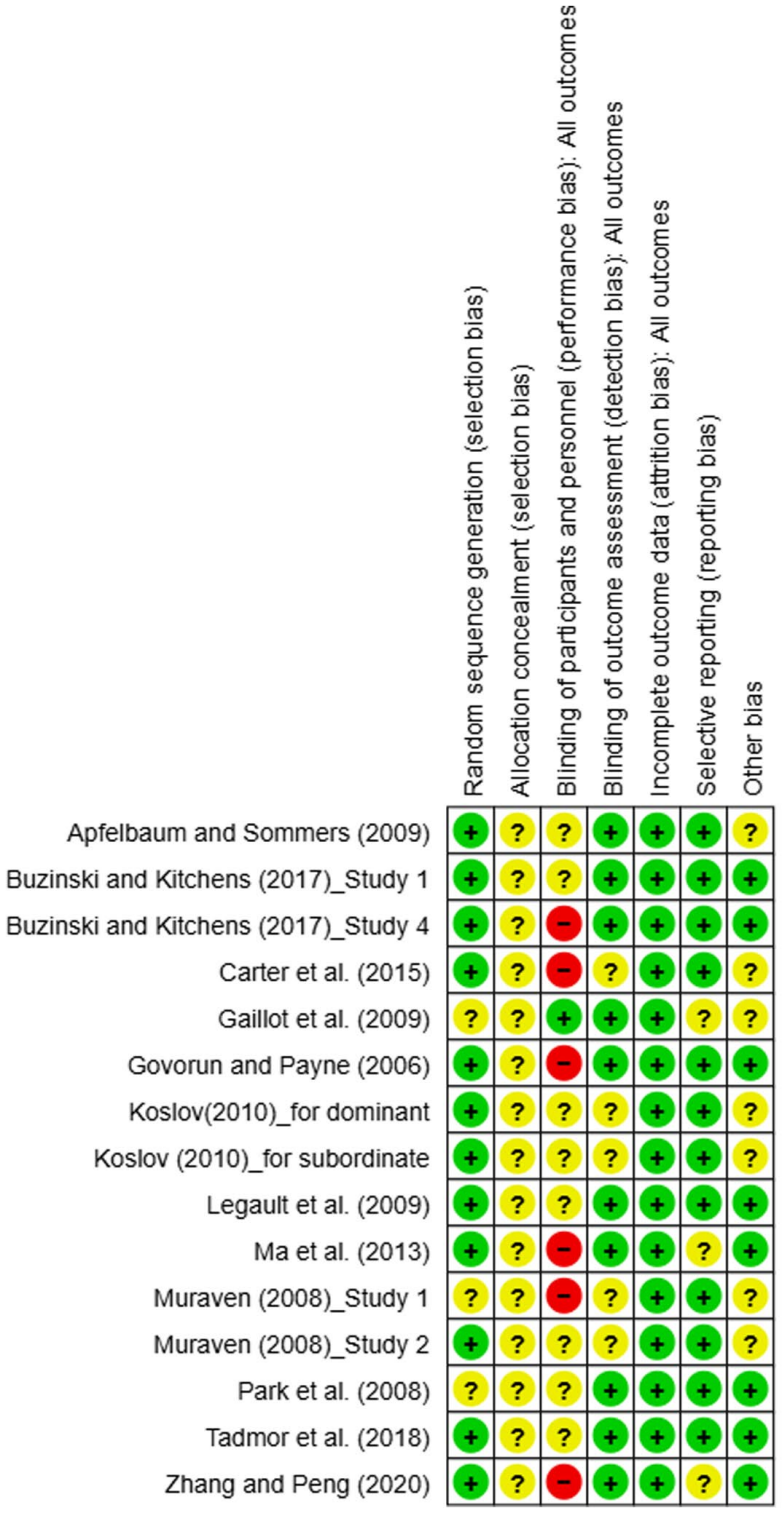
Risk of bias summary for studies on the ego depletion effect on intergroup relations.

Fifteen studies, including 12 journal articles and one PhD thesis, were assessed for risk of bias using Review Manager 5.4. Among these studies, only three did not report the use of random assignment. Additionally, the original publications discussed whether participant allocation was adequately concealed. The completeness of outcome data, selective reporting, and other potential biases were generally judged to be at low risk. Four of the studies utilized blinding. Based on the overall risk of bias assessment, all 15 studies were identified as having a risk of bias. Detailed findings are presented in Figures 5, 6.

#### 3.2.3 The effect of ego depletion on dominant group members

Research involving dominant group members has shown that decreased self-resources can lead to increased prejudiced behaviors in intergroup relations. For example, Govorun and Payne (2006) found that participants who experienced ego depletion exhibited higher levels of controlled racial bias than those in a control group, though both groups displayed similar levels of automatic bias. In a separate study, Ma et al. (2013) demonstrated that depleted participants showed a greater shooting bias compared to individuals in a control group. More recently, Zhang and Peng (2020) reported that depleted participants rated older adults as more forgetful than those in the control group.

Ego depletion can also undermine the positive effects of multicultural experiences in intergroup relations. In a study with Israeli Jewish participants, Tadmor et al. (2018) discovered that when participants reflected on their multicultural experiences, they reduced their support for stereotypes against Arabs. However, this positive effect was only observed when participants were not experiencing ego depletion.

Additionally, research by Gailliot et al. (2009) provided further evidence: when participants’ self-control was increased through manipulated glucose levels, they were better able to suppress stereotypes about subordinate group members. Elevated glucose levels allowed participants to manage their prejudices more effectively. Overall, these findings highlight the critical role of self-control in managing prejudice and stereotypes regarding subordinate group members.

Conversely, research by Apfelbaum and Sommers (2009) suggested that depleted White participants enjoyed their conversations with a Black collaborator more and engaged in discussions about ethnic diversity more openly than those in the control group. Interestingly, both the collaborators and independent Black researchers viewed them as less biased compared to participants in the control group.

Closer examination of the literature highlights the role of the motivational and situational processes. The evidence repeatedly showed the role of MAP, which reflects their desire to seem unbiased (Dunton and Fazio, 1997). Muraven (2008) demonstrated that ego depletion influences only those with a high MAP. Non-depleted participants with a high MAP used fewer negative statements when describing a picture of a Black person (Experiment 1) and highlighted more positive traits when ranking the traits of Black individuals in the community (Experiment 2) compared to depleted participants with the same high MAP. This trend did not appear among participants with a low MAP.

Similarly, Koslov (2010) found that White participants with a strong external motivation to control prejudice tended to engage in overcorrection—exaggeratedly positive behaviors aimed at masking prejudice—when interacting with Black individuals. According to Koslov (2010), such behaviors require self-control, making it challenging for individuals to exhibit excessive positivity when their self-regulatory resources are drained. Consequently, White female students with high motivation to control prejudice demonstrated more overcorrective behavior when they had sufficient self-resources. However, when their resources were depleted, they ceased to display such behaviors toward Black partners (Koslov, 2010).

Research on different groups indicates that ego depletion influences the behavior and attitudes of individuals with low motivation to control prejudice. Legault et al. (2009) found that White participants with low self-determined motivation showed higher levels of implicit discrimination after experiencing ego depletion compared to those in a non-depleted condition. However, depletion did not significantly affect implicit prejudice among participants whose motivation to control prejudice was self-determined. Similarly, Park et al. (2008) studied the role of implicit motivation to avoid prejudice (IMAP), an unconscious goal of maintaining egalitarian beliefs (Glaser and Knowles, 2008). Their findings revealed that while ego depletion did not impact the shooting bias of individuals with high IMAP, it did increase bias among those with low IMAP.

Furthermore, ego depletion can influence people’s MAP. Buzinski and Kitchens (2017; Study 1) reported that the MAP against LGBTQ+ individuals decreased after participants experienced ego depletion. Conversely, this effect was not observed in relation to prejudice against African Americans, possibly due to differences in the measurement tools used. These inconsistent findings highlight the need for more research on the effects of ego depletion on MAP and intergroup relations.

Additionally, social contexts and norms can mitigate the negative effects of ego depletion on the expression of prejudice. In one study, while depleted participants displayed more prejudice towards LGBTQ+ individuals, this effect was absent when there was a strong social pressure to be unbiased (Buzinski and Kitchens, 2017; Study 4).

Studies examining the effect of ego depletion on dominant group members have drawn attention to the necessity of self-control in suppressing prejudice by demonstrating that depleted individuals have difficulty suppressing their prejudices and stereotypes compared to other groups (Govorun and Payne, 2006; Muraven, 2008; Park et al., 2008). The results support Hypothesis 3, indicating that depletion enhances behaviors and attitudes that reinforce hierarchy. These findings align with previous research by Van Berkel et al. (2015), which showed that depletion leads to biased preferences while reducing egalitarian views.

#### 3.2.4 The effect of ego depletion on subordinate group members

Only two studies explicitly looked at the impact of ego depletion on members of subordinate groups. Koslov (2010) demonstrated that, although Black female participants with high rejection sensitivity showed a decreased liking for their White partners in the control condition, this effect was mitigated when self-resources were depleted. In a similar vein, Carter et al. (2015) showed that depleted Black individuals were more likely than the control group to view modern racist attitudes as less biased. This implies that ego depletion may result in a more permissive interpretation of subtle prejudice. These findings suggest that ego depletion may have a substantial impact on how members of subordinate groups perceive and react to prejudice and the dominant group members.

In general, it may seem that ego depletion has typically “positive” effects on members of subordinate group. It prevented Black female students from liking their White partners less (Koslov, 2010). In another study, Black students perceived modern racist attitudes of dominant group members as less prejudiced (Carter et al., 2015). Nevertheless, this interpretation needs attention. According to Carter et al. (2015), if negative behaviors of dominant group members are not attributable to bias, some negative consequences for subordinate group members may occur because attribution to bias is associated with positive outcomes: decreased negative feelings for personal and collective well-being and increased individual and collective self-esteem (Branscombe et al., 1999). This makes it clear that ego depletion does not truly have positive effects on subordinate group members and that its broader impacts need to be examined.

The existing evidence provides partial support for Hypothesis 4, which proposes that subgroup members experiencing ego depletion are more likely to engage in pro-hierarchy behaviors. However, the evidence remains limited, as only a few studies have explicitly examined ego depletion in subordinate group members. In addition, none of these two studies directly measured overt pro-hierarchy behaviors of subordinate group members. Therefore, even the data is consistent with theoretical predictions of Hypothesis 4, further research is needed to confirm the behavioral manifestations of increased compliance with hierarchical structures under conditions of ego depletion.

## 4 General conclusion

Research on ego depletion in intergroup relations has highlighted a reciprocal relationship between intergroup interactions and ego depletion. While intergroup interactions may deplete individuals’ self-regulatory resources, ego depletion may, in turn, influence the nature of intergroup interactions and perceptions. The emergence of ego depletion in intergroup contexts is influenced by an individual’s position within the social hierarchy. For dominant group members, efforts to suppress prejudice during interactions with subordinate group members may lead to ego depletion. Factors such as individuals’ explicit and implicit prejudices, motivation to control these prejudices, and stress levels contribute to this process. This suggests that intergroup interactions do not directly cause ego depletion for dominant group members; instead, depletion occurs when individuals are explicitly required to suppress stereotypes and prejudices against subordinate group members. Regarding SDT, we suggest that hierarchy-attenuating contexts may lead to more depletion for dominant group members than hierarchy-enhancing ones.

For subordinate group members, on the other hand, interactions with dominant group members are often enough to deplete self-resources. Even the mere perception of stereotype threat may result in ego depletion negatively affecting their daily lives. Due to ideological asymmetry, subordinates may perceive more conflict daily. Further studies could explore the role of ideological asymmetry, which suggests a negative relationship between hierarchy-enhancing myths and in-group identification (Levin et al., 1998) on subordinate members’ self-control levels.

Based on these findings, the effect of ego depletion in dominant group members is most significant when the person’s prejudice towards the group with which they interacted was high and intrinsic MAP was low. In the SDT framework, this corresponds to situations in which there is a mismatch between individuals’ tendencies to support hierarchy and the level of support of society. Future research may investigate whether such inconsistency causes the depletion of an individual’s self-resources.

The available findings suggest that ego depletion is not just an individual phenomenon, but also a mechanism that helps sustain existing social hierarchies (Sidanius and Pratto, 1999). Research indicates that ego depletion in intergroup relations affects not only subgroups but also members of the dominant groups (Richeson and Shelton 2003; Govorun and Payne, 2006). However, the reasons for this depletion differ between groups. Dominant group members may experience depletion due to social norms and the desire to avoid appearing prejudiced. As a result of these potential costs, they may choose to avoid intergroup interactions (Meleady et al., 2020; Tesi et al., 2023). This avoidance contributes to the long-term maintenance of existing hierarchical relationships.

On the other hand, the presence of dominant group members can often threaten the identity of subordinate group members, adversely affecting their daily behavior (Berkman et al., 2017). As a result, subgroup members frequently find it difficult to mobilize for collective action or advocate for a new system, which ultimately supports the maintenance of the status quo (Sidanius and Pratto, 1999).

The “cooperative” mechanism becomes increasingly apparent when considering the consequences of ego depletion in intergroup relations. While depleted dominant group members show more prejudiced attitudes and discriminatory behavior, depleted subordinate group members seems to perceive less prejudice and like the dominant group members more. These findings point to the role of ego depletion in forming outgroup favoritism and the maintenance of hierarchical relationships. Studies examining whether high-SDO members of dominant groups experience ego depletion in egalitarian environments more are crucial to understanding the role of ego depletion in everyday social life.

Furthermore, ego depletion may serve as a trigger for self-debilitating behaviors among subordinate group members. Negative actions and failures by these individuals may be attributed to societal processes as well as personal characteristics. Intergroup interactions may become even more debilitating for subordinate group members when societal support for hierarchy-enhancing myths increases, as these myths promote discriminatory behaviors by dominant group members (Aiello et al., 2018; De Oliveira et al., 2012; Ramati-Ziber et al., 2020). Further research may explore the role of ego depletion in the self-debilitating behaviors of the subordinate group members.

System Justification Theory (SJT; Jost and Banaji, 1994), which shares SDT’s view that subordinate group members support hierarchy by demonstrating outgroup bias, may also explain these findings. SJT asserts that people seek both ego and group justification and system justification (Jost and Banaji, 1994). According to SJT, these three basic motives are in harmony for dominant group members but in conflict for subordinate group members (Jost and Burgess, 2000). Therefore, the conflict between these three motives should be more significant for subordinate group members, especially those with a high identification level with the group. This conflict may account for the reduced self-control resources of subordinate group members. Consequently, we argue that subordinate group members with higher levels of identification with the group will experience more cognitive dissonance and need to exercise more self-control to reduce cognitive dissonance. In line with this proposal, for example, Bair and Steele (2010) found that subordinate group members are negatively affected by intergroup interactions when group membership is important to them. Future studies can examine the mediating role of conflicting motivations in the depletion of self-resources in intergroup interactions of subordinate group members within the framework of SJT.

The findings suggest that ego depletion plays an important mediating role in the maintenance of intergroup relations and social hierarchies. However, these findings can be interpreted not only within the framework of the limited resources hypothesis (Baumeister et al., 1998) but also within the context of alternative explanations for ego depletion. For example, from an opportunity-cost perspective (Kurzban et al., 2013), low-status individuals may engage in self-regulation to conform and gain acceptance in intergroup relationships; this may increase their perception of opportunity costs, strengthen their tendency to engage in more rewarding activities, and lead them to engage in self-debilitating behaviors. Regardless of the underlying explanation, the findings suggest that ego depletion can be considered a central mechanism supporting the maintenance of hierarchies and social inequalities.

The current study has some limitations. The first limitation is related to publication bias. As primarily published studies were included in this review, the results reflect only the findings of those studies rather than the overall picture. Considering the current replication crisis related to the ego depletion effect (Carter and McCullough, 2014), this creates a significant limitation. Examining the findings of experimental and quasi-experimental studies constitutes another limitation of the article. Evaluating the relationship between intergroup relations and self-control through other methods will contribute to the generalizability of the results. Furthermore, as this review is only a qualitative synthesis, it does not report the strength of relevant mediators and moderators.

Also, most of the studies were conducted on social hierarchies in the US social context (e.g., divisions between White people and Black people), and other groups (e.g., immigrants-host society) are underrepresented in these studies. Studies with groups from different cultures may provide more detailed information on how ego depletion occurs and how depletion affects intergroup relations.

The ongoing debates about replicating the ego depletion effect have raised concerns about the reliability and reproducibility of findings in subsequent studies. However, as highlighted by Dang (2016), the lack of observed effects may often stem from methodological limitations, such as the researchers’ inexperience and the inadequate strength of the manipulations employed. On the other hand, the standardized assessments of the quality of the studies reviewed indicate low discrepancies between findings, and most effect sizes reported are moderate to large (See Tables 1, 2), which enhances confidence in the evidence presented. Future studies that quantitatively evaluate results through meta-analysis will further contribute to the development of this literature.

In conclusion, although studies on the phenomenon of ego depletion are generally examined independently of intergroup relations theories in the literature, they revealed findings consistent with the basic hypotheses of SDT. Addressing the role of ego depletion in intergroup relations within the basic concepts of intergroup theories and in a multifaceted manner will make valuable contributions to understanding the relationship between self-control and intergroup processes and increase the depth of explanations. Examining this concept using different methodologies will significantly contribute to the reliability of this effect (Inzlicht and Friese, 2019) and to the connection of the ego depletion effect with real-life phenomena such as institutional discrimination.

## Data Availability

The original contributions presented in the study are included in the article/supplementary material, further inquiries can be directed to the corresponding author/s.
